# OVO-like Proteins in Cancer, Immunity, and Inflammation

**DOI:** 10.3390/cells15191755

**Published:** 2026-09-26

**Authors:** Shuxian Huang, Genjian Hu, Hao Lan, Jinfeng Gan

**Affiliations:** 1 Guangxi Key Laboratory of Tumor Immunology and Microenvironmental Regulation, Guilin Medical University, Guilin 541199, China; shuxianhuang@stu.glmu.edu.cn (S.H.);; 2 Guangxi Health Commission Key Laboratory of Tumor Immunology and Receptor-Targeted Drug Basic Research, Guilin Medical University, Guilin 541199, China; 3 Department of Immunology, College of Basic Medicine, Guilin Medical University, Guilin 541199, China

**Keywords:** OVO-like proteins, epithelial–mesenchymal transition, cellular plasticity, metabolic reprogramming, immunity, inflammation

## Abstract

The OVO-like (OVOL) proteins OVOL1–3 are evolutionarily conserved C2H2 zinc-finger transcription factors. Initially identified in *Drosophila* as regulators of germline development, these proteins have since been found to be key determinants of epithelial lineage and differentiation in mammals. Increasing evidence implicates OVOL proteins in human disease, particularly cancer, where they participate in diverse processes including metastasis, metabolic regulation, immunity, and inflammation modulation. In this review, current advances in OVOL-related research are summarized. First, the roles of OVOL proteins in regulating epithelial–mesenchymal transition (EMT), cancer metastasis, and cellular plasticity are addressed. The discussion then extends to emerging evidence for their involvement in metabolic reprogramming, immunity, and inflammation. Finally, open questions and future research directions are discussed.

## 1. Introduction

The *ovo* gene was originally identified in *Drosophila* as a determinant of germline sex [1]. The OVOL family comprises OVOL1–3, which share four conserved C2H2 zinc finger domains and bind the consensus DNA motif (CC/TGTTA), thereby regulating transcription [2]. In humans, *OVOL1*, located on chromosome 11q13.1, and *OVOL2*, located on 20p11.23, are both crucial in the regulation of epithelial differentiation [3,4,5,6]. *OVOL3* maps to chromosome 19q13.12 and is enriched in the testis [1]. A number of research has elucidated the roles of OVOL1/2 in development, immunity, and tumorigenesis; however, OVOL3 remains relatively understudied.

OVOLs regulate diverse cancer-related processes, including EMT [7], metastasis [8,9], metabolic reprogramming [2], and therapy resistance [10], by modulating various downstream targets or pathways. Moreover, emerging roles of OVOLs in shaping the tumor microenvironment (TME) [11] and their correlation with immune cell infiltration [12,13] have been reported. In addition, OVOLs have been demonstrated to be involved in the regulation of multiple inflammatory diseases, including dermatitis [14] and eosinophilic esophagitis (EoE) [15].

Development, immunity, and tumorigenesis all depend on the balance between controlled and dysregulated cellular behavior. These processes are tightly linked through shared molecular signaling networks, in which OVOLs play important roles. This review integrates the roles of OVOL proteins in EMT, cancer metastasis, and cellular plasticity with their emerging functions in metabolic reprogramming, immunity, and inflammation, summarizes the signaling pathways and mechanisms of OVOLs across different diseases, and discusses their clinical relevance.

## 2. OVOLs Maintain Cellular Homeostasis by Regulating Epithelial–Mesenchymal Transition/Mesenchymal–Epithelial Transition

EMT is the reprogramming event by which polarized epithelial cells gain mesenchymal features [16]. EMT and its converse process, the mesenchymal–epithelial transition (MET), are closely associated with various human diseases, including cancer [17]. A canonical function of EMT is to generate migratory cells capable of degrading and traversing the basement membrane and disseminating to distant sites [18]. The dynamic equilibrium between EMT and MET is governed by a class of transcription factors known as MET-inducing transcription factors (MET-TFs), which includes the OVOLs [19,20]. These evolutionarily conserved proteins antagonize EMT-inducing factors [17,21,22].

The relative expression of EMT inducers and OVOLs may regulate hybrid E/M states, leading to either tumor suppression or tumor promotion. Multiple signaling pathways are involved in this network (Figure 1) and are discussed in the following sections.

### 2.1. OVOLs Regulate E/M State Transitions Through the miR-200/ZEB1 Double-Negative Feedback Loop

During cancer progression, miRNAs also play important roles by regulating EMT-TFs [23]. For instance, miR-200 directly suppresses ZEB1/2, whereas ZEB1/2 reciprocally repress miR-200, thereby stabilizing cells in either an E or an M state [24,25]. The double-negative feedback loop between miR-200 and ZEB1/2 acts as a master regulatory switch in EMT [26]. In the epithelial state, the *ZEB1* locus is maintained in a poised chromatin configuration [27] and is directly repressed by OVOL1/2 [28,29,30,31].

In mouse epidermal progenitor cells, Lee et al. [29] demonstrated that Ovol1 and Ovol2 directly repress *Zeb1*, relieving Zeb1-mediated suppression of α-catenin and thereby promoting actin cytoskeleton remodeling and the maturation of cell–cell adhesion. This process maintains epithelial identity by suppressing EMT, thereby ensuring normal epidermal development. In breast cancer, Han et al. [32] found that Ovol2 is predominantly expressed in luminal epithelial cells, whereas Zeb1 is enriched in basal cells. Loss of Ovol2 increases Zeb1 expression, whereas loss of Zeb1 upregulates Ovol2 expression in basal cells. Notably, *Ovol2* knockout alone increases basal-like organoid development, whereas simultaneous knockout of both *Ovol2* and *Zeb1* restores the balance between basal and luminal organoids, indicating that this mutual inhibition loop maintains E/M homeostasis in the mammary epithelium.

Accumulating evidence indicates that EMT operates as a multistep continuum rather than a discrete binary switch [33]. Between the fully epithelial and fully mesenchymal phenotypes lie the E/M phenotypes known as hybrid E/M states. Cells in these hybrid E/M states co-express both epithelial and mesenchymal markers, conferring enhanced tumorigenicity, metastatic potential, and therapy resistance [34,35]. Hong et al. [36] identified two hybrid states between the E and M phenotypes when investigating the relationships among OVOL2, EMT, and ZEB1 by using a systems biology approach. The state closer to E was designated I1, whereas that closer to M was designated I2. These findings collectively support a multistep E–M transition model rather than a binary switch. The OVOL2–ZEB1 mutual inhibition loop governs the establishment and stability of this four-state system. This model is supported by multiple lines of evidence [31,32,37].

Collectively, EMT is highly dynamic, with OVOL2 underpinning its complexity and providing a biochemical basis for cellular plasticity.

### 2.2. The BMP–OVOL1 Positive Feedback Loop Maintains Epithelial Identity by Antagonizing TGF-β Signaling

The TGF-β superfamily signaling pathways, including the TGF-β/Activin and bone morphogenetic protein (BMP) branches, play central roles in regulating E–M plasticity in cancer [38,39,40]. These pathways are tightly regulated. BMP uses receptor-regulated SMADs (R-SMADs; SMAD1/5/8), whereas TGF-β/Activin uses SMAD2/3, and both converge on SMAD4, the common mediator SMAD (Co-SMAD). To ensure signaling specificity, cells employ multilayered regulatory checkpoints that integrate diverse cues into synergistic or antagonistic responses [38]. Fan et al. [41] demonstrated that OVOL1 interacts with SMAD7, a negative regulator of TGF-β type I receptor (TβRI) stability, and prevents its ubiquitination and degradation. Stabilization of SMAD7 promotes TβRI degradation and then attenuates TGF-β signaling, thereby suppressing breast cancer metastasis. Notably, BMP signaling robustly induces OVOL1 expression, which in turn enhances BMP signaling, forming a positive feedback loop. Additionally, the small-molecule compound 6-formylindolo[3,2-b]carbazole (FICZ), an agonist of the aryl hydrocarbon receptor (AhR), has been shown to upregulate OVOL1 expression, thereby partially reversing TGF-β-induced EMT and extravasation in breast cancer cells. Together, these findings establish OVOL1 as a novel suppressor of TGF-β/SMAD signaling that maintains epithelial traits in breast cancer.

### 2.3. OVOL2 Suppresses Wnt Signaling, Thereby Maintaining Epithelial Homeostasis

Colorectal cancer (CRC) is third in incidence in both sexes, and second in global cancer-related death, highlighting its clinical importance [42]. Ye et al. [9] demonstrated that, in CRC, OVOL2 forms a ternary complex with β-catenin/TCF4 by binding both factors. This interaction does not disrupt β-catenin–TCF4 binding or TCF4–DNA association. OVOL2 directly represses the transcriptional activity of the complex, thereby attenuating Wnt signaling and reducing the expression of EMT-associated target genes such as SLUG, ultimately inhibiting EMT and metastasis. Mechanistically, OVOL2 recruits histone deacetylase 1 (HDAC1) to the TCF4/β-catenin complex at the *SLUG* promoter, enhancing local histone deacetylation and chromatin compaction to repress transcription. This HDAC-dependent pathway blocks Wnt-driven EMT and preserves epithelial homeostasis. Thus, disruption of this OVOL2–Wnt axis is associated with progression in CRC, and restoring OVOL2 expression may be a strategy to attenuate CRC progression.

**Figure 1:**
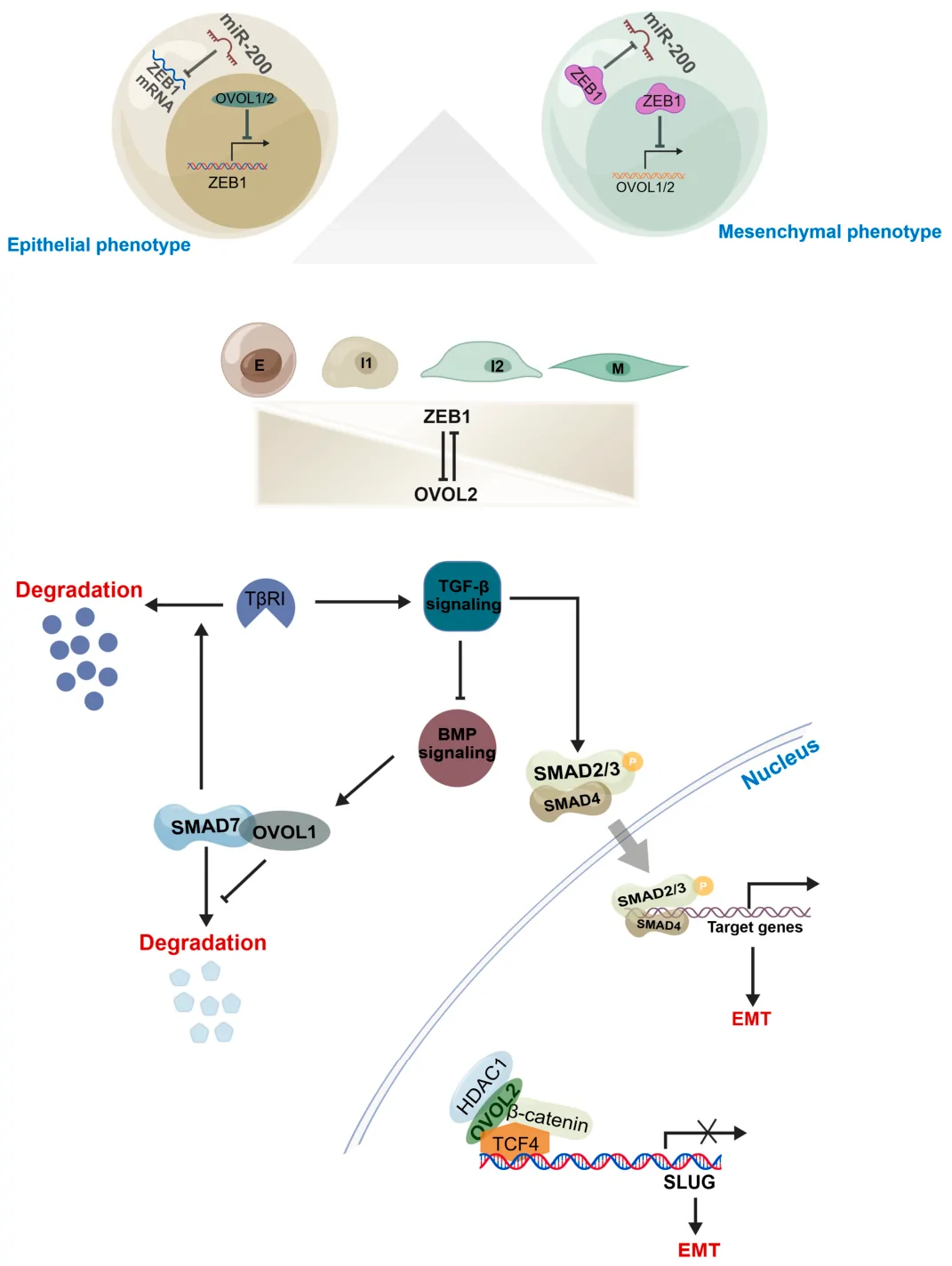
OVOL1/2 mediate multilayered signaling networks regulating epithelial–mesenchymal transition (EMT). The OVOL family (OVOL1/2) functions as an important regulatory hub embedded within hierarchical networks to precisely control EMT and cell-state transitions. Core mechanisms include (1) double-negative feedback loops, in which OVOL1/2 directly repress ZEB1 while the miR-200/ZEB1 circuit serves as a master EMT switch, collectively forming a four-state system (E–I1–I2–M) in which cellular states are determined by the relative strength of pro-EMT signals (e.g., ZEB1) and anti-EMT signals (e.g., OVOL); and (2) integration of multiple signaling pathways, whereby OVOL1 antagonizes TGF-β signaling by stabilizing SMAD7, and OVOL2 inhibits Wnt transcriptional output through interaction with β-catenin/TCF4 and recruitment of HDAC1, thereby maintaining epithelial characteristics at multiple regulatory levels. Created with BioGDP.com [43].

## 3. OVOLs Regulate Cancer Cell Plasticity

Phenotypic plasticity, a hallmark of cancer progression, enables cancer cells to undergo transitions that promote invasion, metastasis, and therapy resistance [44]. OVOLs are important nodes governing these processes (Figure 2). The following sections discuss the mechanisms by which OVOLs regulate cellular plasticity in cancer.

### 3.1. Breast Cancer

It has been established that OVOL1 inhibits cell invasion in breast cancer by promoting TβRI degradation [41]. However, OVOL1’s tumor-suppressive action may cause cancer cells to enter a dormant state [45], a phenotype characterized by enhanced epithelial phenotype (epithelialization) and reversible growth arrest, both of which contribute to therapy resistance, recurrence, and metastasis [46]. Drago-García et al. [45] showed that OVOL1/2, especially OVOL1, are positively regulated by EGF and glucocorticoids and negatively regulated by estrogen and drive the MET through ZEB1 repression and E-cadherin upregulation, inducing dormancy characterized by growth arrest and loss of migration. Mechanistically, OVOL1/2 upregulate C1ORF116, which functions as an autophagy receptor that interacts with GABARAP (a member of the LC3 family) to degrade thioredoxin (TRX), thereby disrupting redox homeostasis. Through a C1ORF116-independent mechanism, OVOL1/2 can also enhance NRF2’s nuclear-to-cytoplasmic translocation and decrease the transcription of its target genes, including glutamate cysteine ligase (GCLC) and GPX4, further impairing antioxidant defense. In addition, they rewire DDR signaling, causing oxidative DNA damage and altered repair. Although these changes enforce dormancy, accumulated ROS-induced damage and genomic instability may trigger reactivation and recurrence. Collectively, OVOL1/2 induce breast cancer cells to enter a dormant state through re-epithelialization via regulation of hybrid E/M states. However, to support the survival of dormant cancer cells, OVOL1/2 promote autophagy and suppress antioxidant capacity, which is toxic and leads to gradual accumulation of DNA damage and new mutations. Once damage exceeds a threshold, the drive for dormancy escape overrides the constraints that maintain dormancy, resulting in relapse.

### 3.2. Hepatocellular Carcinoma

Liver cancer is the third most common cause of cancer-related death worldwide, representing a global health burden [47]. Thus, understanding its molecular basis is critical. Transient receptor potential vanilloid 1 (TRPV1), a nonselective cation channel and capsaicin receptor [48], is involved in promoting oncogenic transformation and cancer progression [49]; however, the underlying mechanisms remain largely unknown. Xie et al. [50] found that TRPV1 deficiency suppresses OVOL2 expression while enhancing that of ZEB1 in hepatocellular carcinoma (HCC), a major subtype of liver cancer. These reciprocal changes coordinately regulate SOX10, driving EMT and promoting invasion and migration. They concurrently enhance tumor-initiating cell expansion, self-renewal, and tumorigenicity, while tight junction proteins are disrupted, compromising cell polarity and barrier function. These findings implicate OVOL2 in HCC metastasis and stemness, although the underlying mechanisms and its regulation by TRPV1 remain to be elucidated.

### 3.3. Esophageal Squamous Cell Carcinoma

Esophageal squamous cell carcinoma (ESCC) comprises approximately 90% of esophageal cancer cases and ranks seventh in global cancer-related deaths [42,51]. This aggressive malignancy grows rapidly, metastasizes to lymph nodes, and is often diagnosed late, leading to poor survival outcomes [52]. Recently, it was shown that OVOL1 functions as a tumor suppressor in ESCC, specifically targeting the *YAP* promoter to suppress transcription, most likely via HDAC-dependent mechanisms, reducing YAP protein levels and downstream signaling and thereby suppressing proliferation, stemness, and metastasis. YAP reintroduction partially reverses OVOL1-mediated suppression [8]. These findings indicate that OVOL1 suppresses ESCC progression through YAP; however, the underlying mechanisms, including the potential regulation of histone acetylation at the *YAP* promoter, warrant further study.

### 3.4. Nasopharyngeal Carcinoma

Nasopharyngeal carcinoma (NPC) is a highly metastatic malignancy [53]; however, the underlying mechanisms involved remain largely unknown. Qi et al. [31] reported that OVOL2 is one of the most significantly downregulated EMT-related transcription factors in metastatic NPC models. In epithelium-like NPC cells, *OVOL2* knockout triggers partial EMT, significantly enhancing cancer stem cell properties and metastatic potential. Conversely, ectopic expression of OVOL2 promotes epithelial differentiation and reduces malignancy by repressing its canonical target, ZEB1. Notably, retinoic acid (RA) upregulates OVOL2 expression in NPC cells. These findings suggest that OVOL2 is involved in NPC metastasis and stemness and can be induced by RA. However, whether RA upregulates OVOL2 expression directly or indirectly remains to be determined.

### 3.5. Bladder Cancer

The rate-limiting enzyme in ketone body catabolism, 3-Oxoacid CoA-transferase 1 (OXCT1), is a key upstream driver of chemoresistance in bladder cancer [54]. Rohena-Rivera et al. [10] found that OXCT1 retains OVOL1 in the cytoplasm, thereby blocking its nuclear repression of target genes. Loss of OVOL1 transcriptional activity promotes cellular dedifferentiation and the acquisition of stem cell-like properties, which may lead to gemcitabine resistance. In addition, the authors identified PPARG, a regulator of fatty acid oxidation (FAO) [55], as a target of OVOL1. These findings raise the possibility that OVOL1 may modulate gemcitabine sensitivity, at least in part, by suppressing PPARG-mediated FAO. Clinically, high expression of OVOL1 target genes is significantly associated with poor prognosis in bladder cancer patients receiving chemotherapy. Thus, OVOL1 subcellular localization may be indicative of chemotherapeutic resistance.

### 3.6. Cutaneous Squamous Cell Carcinoma

Cutaneous squamous cell carcinoma (cSCC) is the second leading type of skin cancer [56,57], with an increasing incidence and a 2–5% risk of metastasis [58,59]. OVOL1 and OVOL2 were found to be enriched in the normal epidermis and hair follicles, as well as in tumors arising from these cell types [60,61]. Notably, OVOL1 expression is upregulated in Bowen’s disease but downregulated in invasive cSCC [60,62]. Ito et al. [62] further found that OVOL1 represses c-MYC and OVOL2 in cSCC. In Bowen’s disease, OVOL1 and nuclear OVOL2 are highly expressed, whereas c-Myc is expressed at low levels. c-Myc is derepressed upon OVOL1 loss in invasive cSCC; OVOL2 is mislocalized to the cytoplasm and loses its function. Thus, the OVOL1–OVOL2 axis may coordinate c-MYC expression and OVOL2 localization to determine skin cancer invasiveness.

### 3.7. Anaplastic Thyroid Carcinoma

Anaplastic thyroid carcinoma (ATC) is a rare yet aggressive thyroid malignancy, which accounts for <5% of cases but >50% of thyroid cancer deaths [63]. Gugnoni et al. [64] identified OVOL2 as a tumor suppressor in ATC. Mechanistically, OVOL2 directly represses the transcription of *RhoU* and *RhoJ*, two Rho GTPase family members, thereby impairing Rho GTPase signaling. This disruption constrains mitotic progression and alters cytoskeletal dynamics, ultimately reducing cancer cell proliferation and invasiveness.

## 4. OVOLs Regulate Cancer Metabolic Reprogramming

Metabolic reprogramming sustains cancer growth by rewiring glucose and lipid metabolism for energy production and biosynthesis. OVOL1/2 play distinct metabolic regulatory roles (Figure 3), which are summarized in the following sections.

### 4.1. Glycolysis

Aerobic glycolysis, also known as the Warburg effect, is a hallmark of cancer and is driven by metabolic reprogramming that sustains proliferation and malignant progression [65]. Zhang et al. [66] reported that in non-small cell lung cancer (NSCLC), OVOL2 suppresses glycolysis by regulating GLUT1 membrane localization. OVOL2 directly binds p65, thereby impeding p300 recruitment and facilitating HDAC1 binding, leading to NF-κB inactivation and reduced GLUT1 membrane localization. p65, in turn, promotes OVOL2 degradation through the ubiquitin–proteasome pathway. These findings position OVOL2 as an important node linking NF-κB signaling to metabolic reprogramming. However, the E3 ligase mediating its turnover remains to be identified. Collectively, OVOL2 suppresses glycolysis by impairing NF-κB signaling, whereas its own stability is regulated by the ubiquitin–proteasome pathway.

### 4.2. Fatty Acid β-Oxidation

Fatty acid β-oxidation (FAO) catalyzes the oxidation of long-chain fatty acids into acetyl-CoA, which then enters the tricarboxylic acid (TCA) cycle to generate ATP. This energy supply is critical for cell growth and also drives tumor progression, metastasis, and drug resistance in diverse cancers [67]. Recently, OVOL2 has been found to block FAO by suppressing JAK/STAT3 pathway in triple-negative breast cancer (TNBC). Mechanistically, OVOL2 represses *JAK1*/*JAK2*/*TYK2* transcription by binding their promoters and reducing STAT3 Tyr705 phosphorylation; it also directly interacts with phosphorylated STAT3 to recruit HDAC1 to the *CPT1A* and *CPT1B* promoters, thereby repressing STAT3-mediated transcription of these rate-limiting FAO enzymes. This lowers ATP, NADPH/NADP+, and GSH/GSSG levels, elevates ROS, and suppresses stemness and tumorigenicity. Ovol2 loss in mice accelerates tumor growth and metastasis, with elevated FAO and ATP levels [68]. Thus, targeting JAK/STAT3 and FAO may offer a therapeutic strategy for OVOL2-low TNBC.

### 4.3. Cholesterol Metabolism

Cholesterol is essential for mammalian cell membranes. Perturbations in cholesterol homeostasis are significantly correlated with cancer progression [69]. A recent study linked OVOL1 to cholesterol metabolism in NSCLC. Feng et al. [70] reported that OVOL1 is upregulated in NSCLC and correlates positively with lymph node metastasis, advanced stage, and poor prognosis. OVOL1 depletion reduces tumor growth and lung metastasis, decreases intracellular cholesterol levels, and downregulates apolipoprotein E (APOE), a key gene involved in cholesterol metabolism. Notably, *APOE* knockdown phenocopies OVOL1 loss by reducing cholesterol and impairing proliferation and migration, whereas APOE overexpression partially rescues these defects. Thus, OVOL1 may drive NSCLC through APOE-mediated cholesterol regulation. Although OVOL1 does not bind directly to the *APOE* promoter, unknown intermediates are likely involved. Elucidating this axis could clarify tumor metabolic reprogramming.

Collectively, OVOL proteins play important regulatory roles in tumor metabolism. OVOL2 maintains a low-glycolysis metabolic state in epithelial cells. Upon loss of OVOL2 function, NSCLC cells relieve glycolytic suppression, whereas breast cancer cells acquire stemness properties through FAO. In NSCLC with high OVOL1 expression, the activation of cholesterol metabolism may represent an alternative metabolic strategy. Thus, within the same cancer type (NSCLC), OVOL1 and OVOL2 exhibit functional divergence in metabolic regulation. The underlying mechanisms warrant further investigation.

## 5. Immune and Inflammatory Regulation Functions of OVOLs

OVOLs are increasingly recognized as regulators of immune and inflammatory microenvironments, potentially influencing immune cell infiltration and macrophage polarization in tumors and controlling epidermal barrier integrity in skin, with implications for skin and other inflammatory diseases. The following sections summarize the mechanisms by which OVOLs regulate immunity and inflammation (Figure 4).

### 5.1. Immune Cell Infiltration

Pancreatic cancer is the sixth most common cause of cancer-related death worldwide and has a dismal prognosis [42]. It was projected to surpass breast cancer in mortality by 2025 [51]. Recently, it was demonstrated that pancreatic tumors with high *OVOL1* expression exhibit reduced infiltration of CD8^+^ T cells, natural killer cells, and cytotoxic lymphocytes, along with increased tumor mutational burden (TMB) [12]. These findings suggest that high *OVOL1* expression may correlate with an immunosuppressive TME, although this association requires experimental validation.

Similar observations have been reported in clear cell renal cell carcinoma (ccRCC). In ccRCC, *OVOL1* and *OVOL2* expression negatively correlate with CD8^+^ T cell infiltration; *OVOL3* expression negatively correlates with CD4^+^ T cell infiltration but positively correlates with neutrophil infiltration. *OVOL2* expression negatively correlates with the infiltration of macrophages and dendritic cells, whereas that of *OVOL1* positively correlates with B cell infiltration [13]. These findings suggest a link between OVOL expression and the ccRCC TME, although they rely primarily on bioinformatic data and warrant experimental validation.

It should be noted that the above results were derived from bioinformatics analysis without experimental validation; thus, whether OVOLs are associated with immune infiltration and how OVOLs impact the infiltration of immune cells remain to be determined.

### 5.2. Macrophage Polarization

Wu et al. [11] provided direct evidence for the involvement of OVOLs in the immune microenvironment. They showed that OVOL2 binds the *IL-10* promoter in macrophages and represses its transcription. Reduced IL-10 disrupts signaling pathways required for M2 polarization, remodeling the TME and diminishing its tumor-promoting activity. This OVOL2-driven cascade suppresses EMT and the migration, invasion, and metastasis of breast cancer cells in vivo. These findings suggest that OVOL2 may participate in shaping the TME.

### 5.3. Cutaneous Immunity

The skin serves as the main defensive barrier against external factors; disruption of its structural integrity predisposes it to inflammatory skin disorders [71]. OVOL1 and OVOL2 also play important roles in maintaining skin immunity. Dragan et al. [72] showed that Ovol1 and Ovol2 cooperatively maintain epidermal barrier integrity in adult mice. Double knockout severely disrupted the barrier, triggering local cutaneous immune activation characterized by increased CD4^+^ T cells and Langerhans cells in skin-draining lymph nodes and elevated γδ T cells in both the epidermis and dermis. Concurrently, inflammatory mediator levels in both skin tissue and peripheral circulation also increased. Mechanistically, Ovol1 and Ovol2 jointly suppress mesenchymal markers to sustain E/M plasticity and repress inflammation-related genes. These findings indicate that OVOL1 and OVOL2 are required for maintaining skin immunity.

### 5.4. Dermatitis

OVOLs are also implicated in inflammatory skin diseases. Atopic dermatitis (AD) is a chronic pruritic inflammatory disorder affecting 10% to 15% of children and 4% to 8% of adults [73] and is associated with atopy, characterized by excessive IgE production [74] and the Th2 cytokines IL-4 and IL-13 [75,76]. Filaggrin (FLG) is critical for skin barrier integrity [77] and is transcriptionally activated by OVOL1 [78]. IL-4 and IL-13 suppress FLG, loricrin (LOR), and IVL in keratinocytes through IL-4Rα signaling [79]. OVOL1 activation promotes its nuclear translocation and upregulates FLG and LOR; however, in AD, IL-4 and IL-13 block OVOL1 nuclear entry, persistently suppressing FLG. IL-13 also inhibits IVL independently of OVOL1, worsening barrier dysfunction [14,78,80]. In barrier-compromised skin, keratinocytes produce thymic stromal lymphopoietin (TSLP), IL-25, and IL-33, which drive the differentiation of Th2 cells and group 2 innate lymphoid cells (ILC2s) and stimulate IL-4, IL-5, and IL-13 production. These cytokines in turn recruit IgE and eosinophils [81]. This establishes a vicious cycle that sustains AD pathology.

Another study showed that skin microbiota dysbiosis inhibits the OVOL1–FLG axis, worsening inflammation. Lee et al. [82] found that commensal bacteria, such as *C. avidum* R CH3 and *S. hominis* R9, activate AhR to upregulate OVOL1 expression. This enhances the transcription of barrier-related genes, including *FLG* and *LOR*, and restores barrier function. In contrast, *S. aureus* suppresses FLG by activating the STAT6/STAT3 pathway via induction of IL-4 and IL-13. Notably, the AhR–OVOL1 axis counters this suppression. AhR dependence was confirmed by silencing *AhR*, which reduced OVOL1 and barrier gene expression and blocked *C. avidum*-mediated repair. In addition, both *S. hominis* R9 and *C. avidum* R CH3 suppress *S. aureus* biofilm formation, implying that their beneficial effects might involve not only AhR–OVOL1-driven barrier repair but also direct biofilm interference.

Collectively, these studies establish an important role for OVOL1 in maintaining skin barrier integrity, and its dysfunction may contribute to the pathogenesis of skin diseases.

### 5.5. Eosinophilic Esophagitis

Eosinophilic esophagitis (EoE) is a chronic inflammatory esophageal disorder linked to genetic factors and early-life environmental exposures [83]. Azouz et al. [15] made several key observations in a recent study of EoE pathogenesis. In the normal esophageal epithelium, OVOL1 localizes to the nucleus and activates *SPINK7*, an antiprotease [84], by binding to its promoter. SPINK7 maintains epithelial barrier integrity and suppresses TSLP production. In EoE, IL-4 and IL-13 block OVOL1 nuclear entry and induce *calpain-14* transcription, which promotes the degradation of cytoplasmic OVOL1. Reduced OVOL1 further attenuates SPINK7, impairing barrier function and increasing TSLP to fuel type 2 inflammation. Collectively, these findings suggest that inflammatory signals may reprogram OVOL1 activity through post-translational modifications or altered subcellular localization.

In summary, OVOL proteins play important roles in EMT/MET, cellular plasticity, metabolism, the TME, immunity, and inflammation. Table 1 summarizes the functions and mechanisms of OVOL, along with the associated evidence contexts.

## 6. Clinical Significance of Dysregulated OVOL Expression

As discussed above, OVOL dysregulation is observed in various malignancies, in which OVOLs play important roles in tumor progression and are associated with clinical outcomes (Table 2).

In lung adenocarcinoma (LUAD), OVOL2 expression correlates with clinical stage and histological grade, is reduced in advanced tumors [85], and predicts poor overall survival (OS) [86]. In lung squamous cell carcinoma (LUSC), OVOL2 is also reduced but is not prognostic [86]. OVOL2 expression is lower in CRC than in adenomas or normal tissue, inversely correlates with progression, and independently predicts poor OS [9,87]. Low OVOL2 expression similarly predicts poor prognosis in HCC [88] and NPC [31]. In ESCC, low OVOL1 mRNA and protein levels predict shorter OS [8]. In breast cancer, OVOL1 declines with increasing tumor grade and predicts reduced relapse-free survival (RFS) [41], and downregulation of OVOL2 similarly correlates with shorter RFS [89].

In other solid tumors, *OVOL1* and *OVOL2* are reduced in ccRCC according to bioinformatic analyses; *OVOL1* loss predicts poor prognosis, whereas *OVOL2* loss correlates with a better outcome [13]. OVOL1 declines during melanoma progression and predicts worse melanoma-specific survival (MSS) and progression-free survival (PFS) [90]. In carcinoma ex pleomorphic adenoma (CXPA), OVOL1 loss is associated with recurrence and aggressiveness, suggesting a role in maintaining a benign phenotype [91].

OVOL upregulation occurs in some cancers. For example, *OVOL1* is overexpressed in pancreatic cancer and predicts poor OS, disease-specific survival (DSS), and progression-free interval (PFI). Additionally, its promoter hypomethylation correlates with adverse outcomes [12]. Similarly, OVOL1 is significantly upregulated in NSCLC and predicts poor prognosis [70]. Bioinformatic analysis shows that *OVOL3* is upregulated in ccRCC and predicts an unfavorable prognosis [13].

Overall, OVOL dysregulation is frequent across cancers, supporting the prognostic value of OVOLs. However, their prognostic value in cancer patients requires validation in additional independent cohorts, particularly prospective ones.

**Table 2:** Clinical significance of dysregulation of OVOL expression in cancer.

OVOLs	Cancer Types	Methods	Expression	Clinical Relevance	References
OVOL1	Breast cancer	IHC, bioinformatic analysis; retrospective analysis	Downregulation	Predicts reduced RFS	[41]
	NSCLC	IHC, bioinformatic analysis; retrospective analysis	Upregulation	Predicts poorer OS	[70]
	ESCC	IHC, Bioinformatic analysis; retrospective analysis	Downregulation	Predicts poorer OS	[8]
	Pancreatic cancer	IHC, bioinformatic analysis; retrospective analysis	Upregulation	Predicts poorer OS, DSS, and PFI	[12]
	ccRCC	Bioinformatic analysis; retrospective analysis	Downregulation	Predicts poorer OS	[13]
	Cutaneous melanoma	IHC; retrospective analysis	Progressive downregulation	Predicts poorer DSS and PFS	[90]
	CXPA	IHC; retrospective analysis	Downregulation	Associated with tumor recurrence; differentiates between benign and malignant diseases	[91]
OVOL2	LUAD	IHC; retrospective analysis	Downregulation	Associated with higher T stage, TNM stage, and histological grade; predicts poorer OS	[85,86]
	LUSC	IHC; retrospective analysis	Downregulation	Not related to OS	[86]
	CRC	IHC; retrospective analysis	Downregulation	Predicts poorer OS	[9,87]
	HCC	IHC; retrospective analysis	Downregulation	Predicts poorer OS	[88]
	Breast cancer	Bioinformatic analysis; Retrospective analysis	Downregulation	Predicts poorer RFS	[89]
	NPC	IHC; retrospective analysis	Downregulation	Predicts poorer OS	[31]
	ccRCC	Bioinformatic analysis; retrospective analysis	Downregulation	Predicts better OS	[13]
OVOL3	ccRCC	Bioinformatic analysis; retrospective analysis	Upregulation	Predicts poorer OS	[13]

## 7. Discussion and Perspectives

As discussed, OVOLs show prognostic potential across multiple cancers, providing an experimental basis for their application in prognostic monitoring, though their prognostic value requires further investigation. Moreover, OVOL1 and OVOL2 can be induced by FICZ and RA, respectively, whereas geniposide, a component of Gardenia jasminoides, upregulates OVOL3, which activates oxidative phosphorylation and suppresses osteosarcoma progression by directly binding to and activating the *Ndufs8* promoter [92]. These observations suggest that OVOL expression can be induced by pharmaceutical reagents. However, the evidence merely indicates that OVOLs act downstream of these reagents and does not establish that OVOLs are themselves druggable. The development of safe agonists that directly target OVOLs will require further investigation.

Notably, OVOLs can be tumor-suppressive or -promoting depending on the context, highlighting the need to understand their functional plasticity. This plasticity may arise from their cooperation with lineage-specific coregulators that vary across cancer types, as well as from post-translational modifications (e.g., phosphorylation, ubiquitination, and PARylation) that may also affect the transcriptional regulatory functions of OVOLs. In addition, the localization of OVOL proteins also affects their function, and the mechanisms that determine OVOL proteins localization, as well as the cytoplasmic function of OVOL proteins, remain unclear. Experimental approaches such as tissue-specific knockout models, single-cell sequencing, and spatial transcriptomics may help clarify the mechanisms underlying this duality.

As noted, OVOL2 regulates macrophage polarization, OVOL1 is involved in dermatitis and EoE, and OVOLs are associated with immune cell infiltration in the TME. Thus, the interplay between OVOLs and the immune system also represents an important direction for future research.

Although OVOL2 is considered a regulator of hybrid E/M states, most recent studies on OVOL in cancer cell EMT merely detect classical epithelial and mesenchymal markers, which label either the initial epithelial or the terminal mesenchymal state. Partial EMT markers or techniques such as single-cell sequencing are therefore required to further demonstrate the role of OVOL in regulating the hybrid E/M state.

It should be noted that the review’s summary of progress on the OVOL family draws predominantly on studies of OVOL1 and OVOL2, while OVOL3 remains comparatively understudied. This may be attributable to the fact that *OVOL3* ESTs were initially isolated from testis libraries, and the gene was therefore considered to be mainly expressed in the testis [1,93]; however, recent studies have shown that OVOL3 is also expressed in other cells and tissues [13,92,94]. Nevertheless, these studies are often limited to expression-based analyses, with little functional validation. Future studies are needed to investigate the functions and mechanisms of OVOL3 in pathological or disease states and to determine whether the functions and mechanisms identified in OVOL1/OVOL2 are conserved in OVOL3.

## 8. Conclusions

In conclusion, despite the limited number of studies, OVOLs have been suggested to play important roles across diseases. Elucidating their regulatory networks will facilitate the development of targeted therapies for clinical translation.

## Figures and Tables

**Figure 2:**
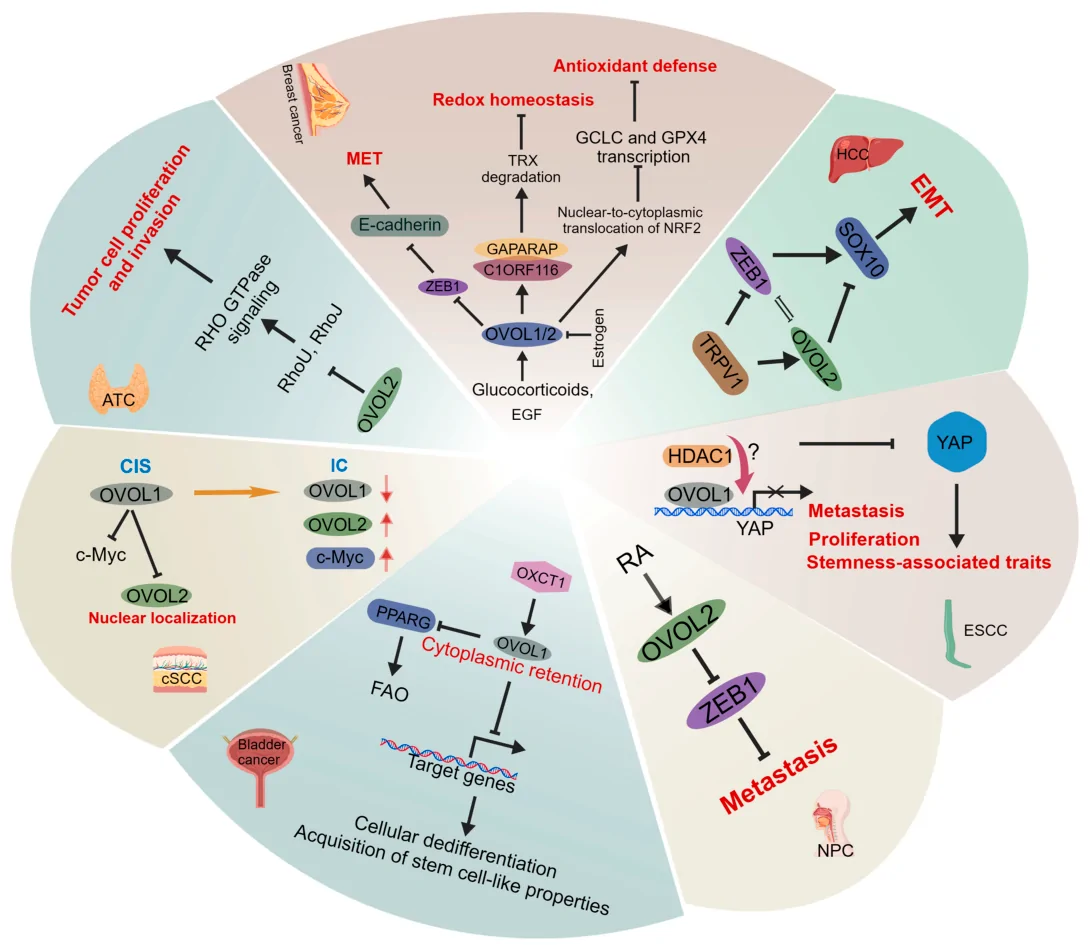
OVOL1/2 act as important regulators of cancer cell plasticity. OVOLs orchestrate cancer cell plasticity across breast, hepatocellular, esophageal squamous cell, nasopharyngeal, bladder, and anaplastic thyroid carcinomas through various signaling cascades, while their own expression is in turn governed by distinct regulatory factors. In cutaneous squamous cell carcinoma, OVOL1 plays a role in cancer progression and functions as a repressor of c-Myc and OVOL2. Downregulation of OVOL1 or aberrant localization of OVOL2 is associated with aggressive phenotypic transformation. CIS, carcinoma in situ (Bowen’s disease); IC, invasive cSCC. Created with BioGDP.com [43].

**Figure 3:**
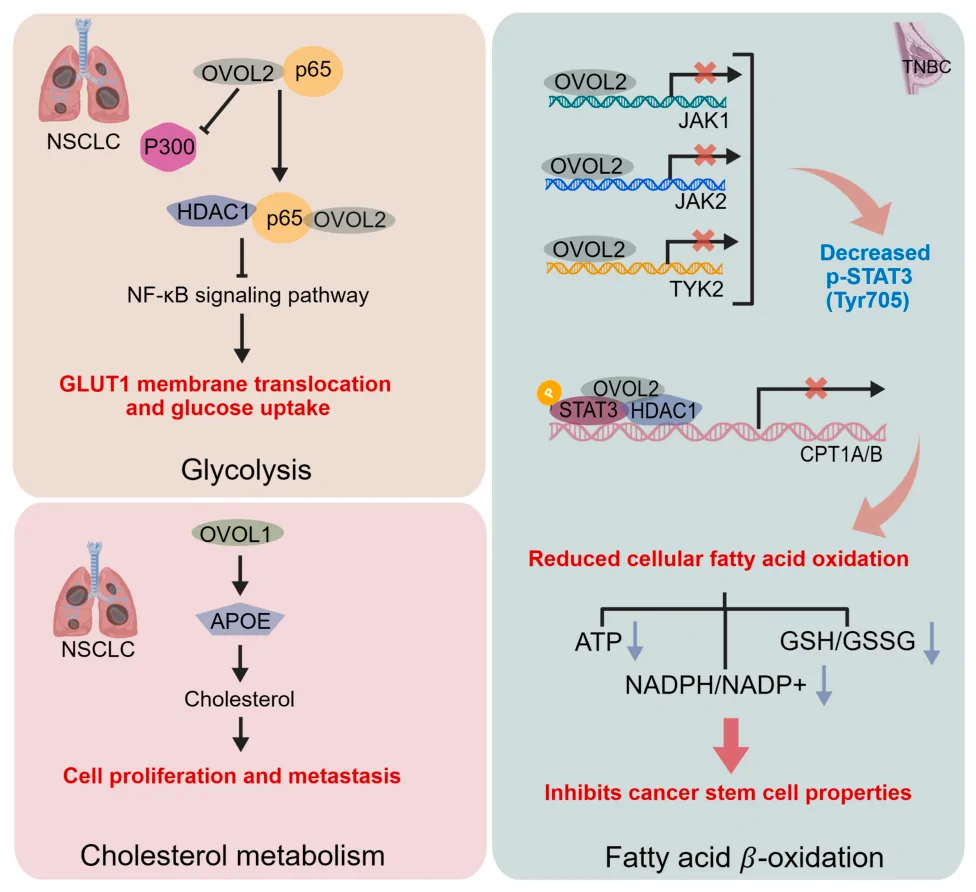
OVOL1/2 in cancer metabolic reprogramming. OVOL1/2 establish a multilayered metabolic regulatory network through suppressing NF-κB signaling, negatively regulating the JAK/STAT3 axis, and modulating APOE expression. Deregulation of OVOL1/2 expression promotes metabolic reprogramming, thereby enhancing cancer cell proliferation, maintenance of stemness, metastasis, and disease progression. Created with BioGDP.com [43].

**Figure 4:**
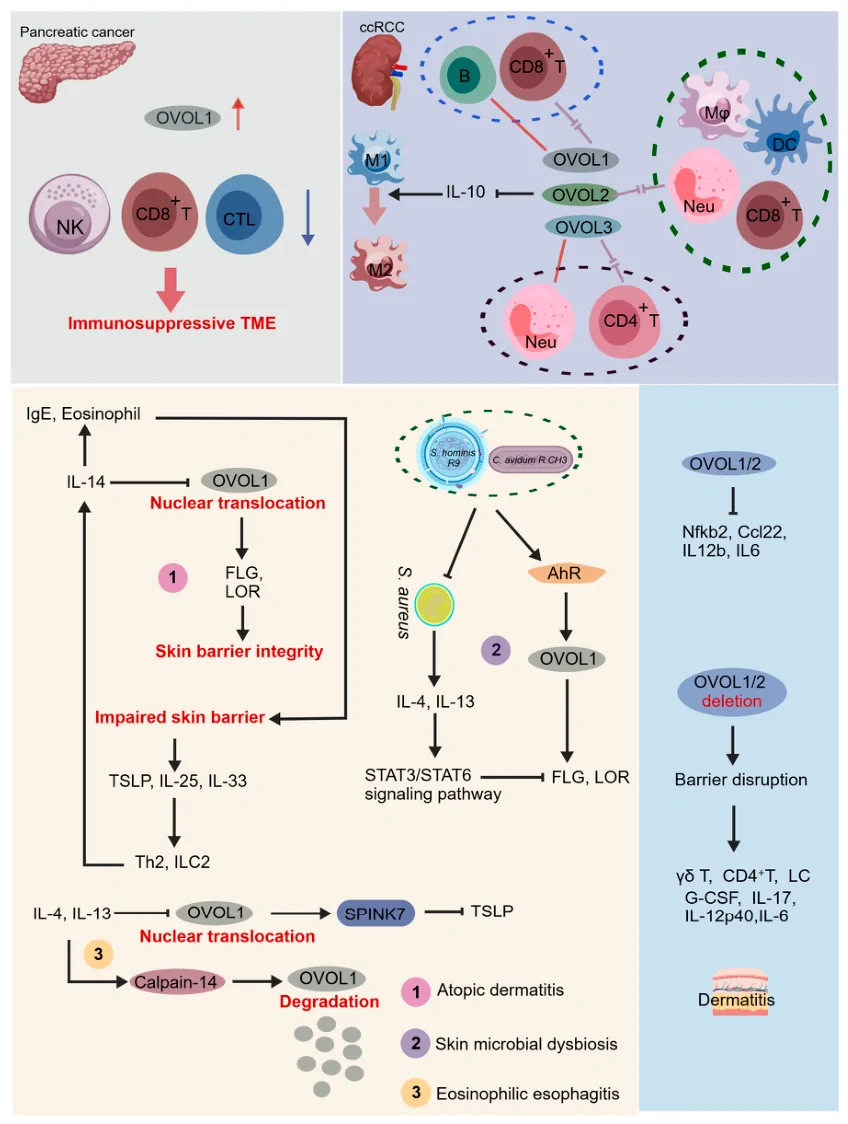
The multifaceted roles of OVOLs in immune and inflammatory regulation. The OVOL family regulates macrophage polarization, inflammatory cytokine expression, and skin barrier protein synthesis, and correlates with immune cell infiltration, thereby playing important roles in shaping the tumor immune microenvironment and inflammatory skin diseases. Dysregulation of OVOL may lead to the establishment of immunosuppressive microenvironments, skin barrier dysfunction, and inflammatory diseases. NKs, natural killer cells; CTLs, cytotoxic lymphocytes; Neu, neutrophils; DCs, dendritic cells. Created with BioGDP.com [43].

**Table 1:** Functions and mechanisms of OVOL in different contexts.

Functional Categories	OVOLs	Study Subjects	Mechanisms	Experimental Models	Biological Effects	References
EMT/MET	OVOL2	Mammary gland	OVOL2 and ZEB1 suppress each other	Basal mammary epithelial cells (MECs) with *Ovol2* or *Zeb1* deletion; *Zeb1* knockout mice	Maintains E/M homeostasis	[32]
	OVOL1	Breast cancer	Promotes the degradation of TβRI	Cells with OVOL1 overexpression and knockdown, and zebrafish and mice injected with these cells	Suppresses breast cancer metastasis	[41]
	OVOL2	CRC	Inhibits Wnt signaling	OVOL2-overexpressing cell xenograft mice; conditional *Ovol2* knockout *Apc*^min^ mice	Inhibits CRC EMT and metastasis	[9]
Cancer cell plasticity	OVOL1/2	Breast cancer	Upregulates C1ORF116 and promotes NRF2 translocation from the nucleus to the cytoplasm	Cells overexpressing OVOL1 or OVOL2 plated on a 3D extracellular matrix; an OVOL1-overexpressing cell xenograft mice; *MMTV*^PyMT/+^/*Ovol1*^tm1a(KOMP)Wtsi^ mice	Promotes breast cancer cell dormancy	[45]
	OVOL2	HCC	OVOL2 is upregulated by TRPV1 to suppress SOX10	*Trpv1* knockout mice	TRPV1-induced OVOL2 suppresses HCC EMT	[50]
	OVOL1	ESCC	Suppresses YAP expression	OVOL1 overexpression and knockdown cells; OVOL1 overexpression and knockdown xenograft mice	Inhibits ESCC cell proliferation, stemness, and metastasis	[8]
	OVOL2	NPC	Suppresses ZEB1 expression	OVOL2 overexpression and knockout cells; *OVOL2* knockout xenograft mice	Inhibits NPC cell metastasis	[31]
	OVOL1	Bladder Cancer	OXCT1 retains OVOL1 in the cytoplasm, inhibiting its transcriptional activity	*OXCT1* knockout cells; gemcitabine-treated cells	OXCT1 retains OVOL1 in the cytoplasm and promotes gemcitabine resistance	[10]
	OVOL1	cSCC	Suppresses the expression of c-MYC and OVOL2	Cells transfected with OVOL1 siRNA	Not available	[62]
	OVOL2	ATC	Suppresses the expression of RhoU and RhoJ	OVOL2-overexpressing cells	Inhibits ATC cell proliferation and invasiveness	[64]
Metabolic reprogramming	OVOL2	NSCLC	OVOL2 interacts with p65 to inactivate NF-κB and reduce GLUT1 membrane localization	OVOL2 overexpression and knockdown cells; OVOL2 overexpression and knockdown xenograft mice	Suppresses glycolysis and tumor growth	[66]
	OVOL2	TNBC	Suppresses JAK/STAT3 pathway	OVOL2 overexpression and knockdown cells; *PyVT*-*Ovol2* knockout mice	Inhibits TNBC cell FAO and stemness	[68]
	OVOL1	NSCLC	Upregulates APOE expression	*OVOL1*-knockdown cells	Promotes cholesterol metabolism, proliferation, and metastasis	[70]
Immunity and inflammation	OVOL2	Breast cancer	Suppresses IL-10 expression	RAW264.7 cells with Ovol2 overexpression and knockdown; BALB/c mice co-implanted with Ovol2-overexpressing Raw264.7 cells and 4T1 cells	Inhibits macrophage M2 polarization and breast cancer cell metastasis	[11]
	OVOL1/2	Skin immunity	Suppresses the expression of EMT-associated and inflammatory genes	*Ovol1* and *Ovol2* double knockout mice	Ovol1/2 loss triggers skin immune activation and impairs the skin epidermis	[72]
	OVOL1	Atopic dermatitis	IL-4 blocks OVOL1 nuclear transport to suppress FLG expression	Normal human epidermal keratinocytes with OVOL1 overexpression and knockdown; an in vitro AD skin model	Regulates atopic dermatitis progression	[78]
	OVOL1	Skin microbiome dysbiosis	*C. avidum* R CH3 and *S. hominis* R9 upregulate OVOL1 to increase FLG and LOR expression.	HaCaT cells co-cultured with skin microbiomes	Restores skin barrier	[82]
	OVOL1	EoE	Upregulates SPINK7 expression	*OVOL1* knockout cells	Depletion of OVOL1 impairs epithelial barrier	[15]

## Data Availability

No new data were created or analyzed in this study. Data sharing is not applicable to this article.

## Abbreviations

The following abbreviations are used in this manuscript:

OVOLs	OVO-like proteins
EMT	Epithelial–mesenchymal transition
MET	Mesenchymal–epithelial transition
R-SMADs	Receptor-regulated SMADs
Co-SMAD	Common mediator SMAD
TβRI	TGF-β type I receptor
CRC	Colorectal cancer
HDAC1	Histone deacetylase 1
TRX	Thioredoxin
GCLC	Glutamate cysteine ligase
TRPV1	Transient receptor potential vanilloid 1
HCC	Hepatocellular carcinoma
ESCC	Esophageal squamous cell carcinoma
NPC	Nasopharyngeal carcinoma
RA	Retinoic acid
OXCT1	3-Oxoacid CoA-transferase 1
FAO	Fatty acid oxidation
CSCC	Cutaneous squamous cell carcinoma
ATC	Anaplastic thyroid carcinoma
NSCLC	Non-small cell lung cancer
TCA	Tricarboxylic acid
TNBC	Triple-negative breast cancer
APOE	Apolipoprotein E
TMB	Tumor mutational burden
TME	Tumor microenvironment
ccRCC	Clear cell renal cell carcinoma
AD	Atopic dermatitis
FLG	Filaggrin
LOR	Loricrin
TSLP	Thymic stromal lymphopoietin
ILC2s	Group 2 innate lymphoid cells
EoE	Eosinophilic esophagitis
LUAD	Lung adenocarcinoma
OS	Overall survival
LUSC	Lung squamous cell carcinoma
RFS	Relapse-free survival
PFS	Progression-free survival
CXPA	Carcinoma ex pleomorphic adenoma
MSS	Melanoma-specific survival
DSS	Disease-specific survival
PFI	Progression-free interval
